# Environmental regulation and carbon emission efficiency: Evidence from pollution levy standards adjustment in China

**DOI:** 10.1371/journal.pone.0296642

**Published:** 2024-02-01

**Authors:** Yi He, Xiang Zhang, Qinghua Xie

**Affiliations:** 1 Work Committee for Offices Directly Under Chongqing Municipality, China; 2 Accounting School, Chongqing University of Technology, Chongqing, China; 3 Guangdong Western Digital Intelligence Accounting Development Research Center, Zhanjiang University of Science and Technology, China; Qufu Normal University, CHINA

## Abstract

China’s economy experienced great growth, which also induces large carbon emission. Facing the target of “Carbon peak, Carbon neutrality” in China, it is vital to improve the carbon emission efficiency. Employing the spatial Difference-in-Differences model, this paper investigates the impact of environmental regulation on carbon emission efficiency with a quasi-natural experiment of Pollution Levy Standards Adjustment in China. Our empirical results show that the environmental regulation can significantly improve the carbon emission efficiency. moreover, two impact channels are explored: green innovation and industrial upgrading. More specifically, the green innovation increases with environmental regulation, and the increased green innovation improves carbon emission efficiency. The industry upgrading increases with environmental regulation, and the increased industry upgrading improves carbon emission efficiency. Finally, in terms of city heterogeneity, we find that the impact of environmental regulation will be more pronounced for larger cities and resource-based cities. Our findings suggest that the environmental regulation must be enhanced for both smaller cities and non-resource-based cities. Moreover, to promote the green innovation of firms, since green innovation is risky and costly, governments should provide more subsidies or grants on corporate green technologies, thus firms will be motivated to invest in green technologies to reduce carbon emission.

## 1. Introduction

The rapid growth of greenhouse gas emissions has caused serious issues on the sustainable growth of the world [1, 2]. China has experienced great economic growth for the last a few decades, however, the tremendous growth also leads to high carbon emission. China’s carbon emission has become the largest over the world, total carbon emission has increased from 1.419 billion tons in 1978 to 9.899 billion tons in 2020 [3]. This means that China faces enormous pressure to reduce emissions. Facing the pressure of carbon reduction, Chinese government proposes the “Carbon peak, Carbon neutrality” goal aiming at carbon emission reduction.

Numerous studies investigate the carbon emission reduction in China [4, 5]. However, in current stage, Chinese economy is still experiencing high growth, there exists great pressure on the reduction of total carbon emission, thus, improving the carbon emission efficiency is much more important. Previous studies concerning carbon emission efficiency generally focus on innovative technologies and green innovation [6–14]. However, the impact of environmental regulation is ignored in current literature, to fill the gap, this paper attempts to investigate the impact of environmental regulation on carbon emission efficiency with a quasi-natural experiment in China.

At early stage, China’s environmental regulation mainly consists in command-and-control, such as carbon emissions trading and carbon tax, however, the effect on emission reduction is weak, and the cost is high and unsustainable. Under the pressure of emission reduction, China tends to explore the market-based mechanism to reduce carbon emission. Moreover, pollution monitoring and administrative controls are proved as the main causes of environmental quality improvements. However, the impact of pollution levy regulation has rarely been examined in previous studies, which has been implemented in China for a long time.

The implementation of pollution levy system has been launched since 1980s. At early stage, firms will only be charged for excess pollution, and the pollution levy fee is relatively low, firms are not motivated for pollution governance. In 2007, State council issued “the Comprehensive work plan for energy conservation and emission reduction”, firms will be charged as they have pollution emission, and it required higher pollution levy standards. As a result, from 2007 to 2013, 12 provinces or municipalities in China gradually doubled the levy standards from the original 0.63 yuan/kg to 1.26 yuan/kg.

This paper takes the Pollution Levy Standards Adjustment (PSLA) in China as a quasi-natural experiment. Considering the spatial correlation, the spatial Difference-in-Differences (DID) method is applied. To measure the carbon emission efficiency, the extended SFA model is used [15]. This method can separately reveal the time-varying inefficiency, time-invariant inefficiency, and urban heterogeneity in the residuals at the meantime. As a result, this paper investigates the impact of PSLA on carbon emission efficiency with spatial DID method. Moreover, two impact channels are also investigated: green innovation channel and industry upgrading channel. Finally, two features of city heterogeneity are considered: size and resource.

This paper contributes to current literature in the following aspects. First, this paper investigates the impact of environmental regulation on carbon emission efficiency. Current studies generally focus on the impact of innovative technologies and green innovation on carbon emission efficiency. In terms of environmental regulation, previous literature generally focuses on command-and-control environmental regulations, such as carbon emissions trading and carbon tax [16, 17]. However, the impact of Pollution Levy policy, which is direct punishment on firm’s pollutant emission, has never been investigated. Meanwhile, to better identify the causal inference, this paper employs a quasi-natural experiment: the Pollution Levy Standards Adjustment in China. Second, to consider the spatial correlation, this paper applies spatial difference-in-difference (DID) method for estimation instead of traditional DID model [18, 19]. Traditional DID method only considers the direct effect of PSLA, however, carbon emission features spillover effect among different cities, which can be shown by spatial DID method. Hence, the results estimated through spatial DID model reduce the interference of spatial factors, which can more reliably assess the impact of government innovation support on carbon emission efficiency. Third, two impact channels are investigated: green innovation channel and industry upgrading channel. The green innovation increases with the PLSA implementation, and the in-creased green innovation improves carbon emission efficiency. The industry upgrading increases with the PLSA implementation, and the increased industry upgrading im-proves carbon emission efficiency. Finally, the city heterogeneity is considered, we find that the impact of PLSA will be more pronounced for larger cities and resource-based cities.

The reminder of this study is organized as follows: Section 2 provides the literature review, the policy background and research hypothesis are presented in Section 3, Section 4 shows the methods and data, empirical results are analyzed in Section 5, Section 6 presents the robustness check, the discussion is presented in Section 7, the conclusion and policy implications are shown in Section 8.

## 2. Literature review

The measurement of carbon emission efficiency has been widely studied in prior literature. Numerous research employ input-output ratio, specifically, the economic output scaled by the carbon emission is used [20–23], carbon emission efficiency is higher as this indicator is larger. However, the weakness of this indicator lies in the ignorance of some potential factors that may impact the economic output, including industry structure and labor force [23]. To overcome this problem, two methods are introduced, including Data envelopment analysis (DEA) and Stochastic Frontier Approach (SFA) [21, 23–27]. The concept of these two methods lies in the difference between input and output, more specifically, carbon emission efficiency is computed by the gap between actual carbon emission and expected carbon emission [24, 28–31]. However, these measures are biased due to unobserved heterogeneity among cities, and it differs across different sample size. The measurement error can be ignored in small sample, but it will lead to large estimation bias in large sample [21, 23]. To relieve this problem, an extended SFA model is proposed [21]. With this model, time-varying features, time-invariant features, and city-level heterogeneity in the residuals can be separated simultaneously.

Another trend of studies concentrates on the determinants of carbon emission efficiency, innovative technologies and green innovation have been proved to have great impact on carbon emission performance. Prior literature study the improvement of carbon emission efficiency with new technologies in the manufacturing process, including clean technology and industrial robots [6–9]. In addition, information technology has experienced great development in China, and it has been embedded in manufacturing process and governance activities for large number of firms, evidence also show that digital transformation also improves carbon emission efficiency [10–12]. Due to intense environmental regulation in China, numerous firms increase their investment in green innovation, and it is proved that the investment in green innovation can significantly improve carbon emission efficiency of firms [13, 14]. However, the cost of green innovation is extremely high, and it does not bring higher excess returns for firms, thus, the green investment cannot be achieved solely by firms, the role of governments is extremely important in energy saving and emission reduction [32–34]. Therefore, the subsidies and policy support granted from governments are vital for green innovation of firms in reducing the risk of R&D activities [35, 36].

In addition to innovative technologies and green innovation, environmental regulations also have great impact on carbon emission. Numerous policies are implemented by Chinese government to reduce industrial carbon emissions. Among those, command-and-control environmental regulation led by the government still dominates. Although market-based tools such as carbon emissions trading and carbon tax have produced significant emissions reduction benefits [16, 17], these benefits have been achieved under the supervision and deterrence of administrative orders and environmental regulations [37]. Most scholars emphasize pollution monitoring and administrative controls as the main causes of environmental quality improvements [38, 39]. Empirical studies based on different countries such as the United States, European Union countries, India, and China have shown that command-and-control environmental regulation is an important driver for reducing environmental pollution and carbon emissions [40–43].

It can be seen that most studies focus on the impact of green technologies, besides, environmental regulations also show great impact on carbon emission performance. However, previous literature only studies the impact of command-and-control environmental regulation or market-based regulation policies, the Pollution Levy policy has never been investigated, this policy presents the direct punishment on firm’s pollutant emission, and the pollution levy standard is adjusted in 2007, which can be employed as a quasi-natural experiment for causal identification. Thus, this paper attempts to investigate the impact of pollution levy standards adjustment on carbon emission efficiency.

## 3. Policy background and research hypothesis

### 3.1. Policy background

Among numerous environmental regulation policies, the implementation of pollution levy system is quit longer and with larger range. At the early stage, the pollution levy fee is relatively low and only excess pollution emission will be charged, firms are not motivated for pollution governance. In 2007, State council issued “the comprehensive work plan for energy conservation and emission reduction”, all pollution emission will be charged and higher pollution levy standards are required. As a result, from 2007 to 2013, 12 provinces or municipalities in China gradually doubled the levy standards from the original 0.63 yuan/kg to 1.26 yuan/kg. The detail of pollution levy standards adjustment is presented in Table 1.

**Table 1:** The detail of pollution levy standards adjustment from 2007 to 2013.

Region	Pollution levy standards adjustment plan (Yuan/kg)
Jiangsu	The pollution levy is raised to 1.26 starting from July 2007
Anhui	The pollution levy is raised to 1.26 in three years, it is 0.84 from January 2008
Hebei	The pollution levy is raised to 1.26 in two years, it is 0.96 from July 2008
Shandong	The pollution levy is raised to 1.26 starting from July 2008
Neimenggu	The pollution levy is raised to 1.26 in two years, it is 0.95 from July 2008
Guangxi	The pollution levy is raised to 1.26 in two years, it is 0.95 from January 2009
Shanghai	The pollution levy is raised to 1.26 starting from January 2009
Yunnan	The pollution levy is raised to 1.26 in two years, it is 0.95 from January 2009
Guangdong	The pollution levy is raised to 1.26 starting from April 2010
Liaoning	The pollution levy is raised to 1.26 starting from August 2010
Tianjin	The pollution levy is raised to 1.26 starting from December 2007
Xinjiang	The pollution levy is raised to 1.26 starting from August 2012

### 3.2. Research hypothesis

First, the PSLA can impact the carbon emission through the green innovation channel. The PSLA will immediately increase the cost of pollutant emission, thus, to reduce cost, firms are motivated in green innovation, they will invest more in emission reduction and other green technologies [44]. The investment in green innovation may conduct two consequences: first, the carbon emission will be reduced due to green technologies [13, 14]; second, the innovation activities may lead to higher output [45, 46]. As a result, the carbon emission efficiency will be improved. Based on the arguments above, we propose the first hypothesis:

H1: The environmental regulation will stimulate the green innovation, and the increased green innovation will improve the carbon emission efficiency.

Second, the PSLA can impact the carbon emission through industry upgrading channel. The increased pollution levy may cause another effect: the escaping effect. due to the increased cost in pollution cost, firms in high pollution industries may leave this city and move to other cities with lower pollution levy. At the meantime, when the enforcement of environmental regulation is high, governments will prefer firms from tertiary industries to invest in the cities [47]. As a result, the PSLA can also promote industrial upgrading. Industrial upgrading leads to a decrease in the share of energy inputs in enterprise production and an increase in the value added of products [48]. Therefore, firms can achieve higher output with lower resource inputs, which leads to the improvement of carbon emission efficiency. Based on the above analysis, we propose the second hypothesis.

H2: The environmental regulation will promote the industry upgrading, and the carbon emission efficiency will increase with the industry upgrading.

Overall, both channels will lead to a positive correlation between environmental regulation and carbon emission efficiency, that is, environmental regulation will improve the carbon emission efficiency.

**Fig 1:**
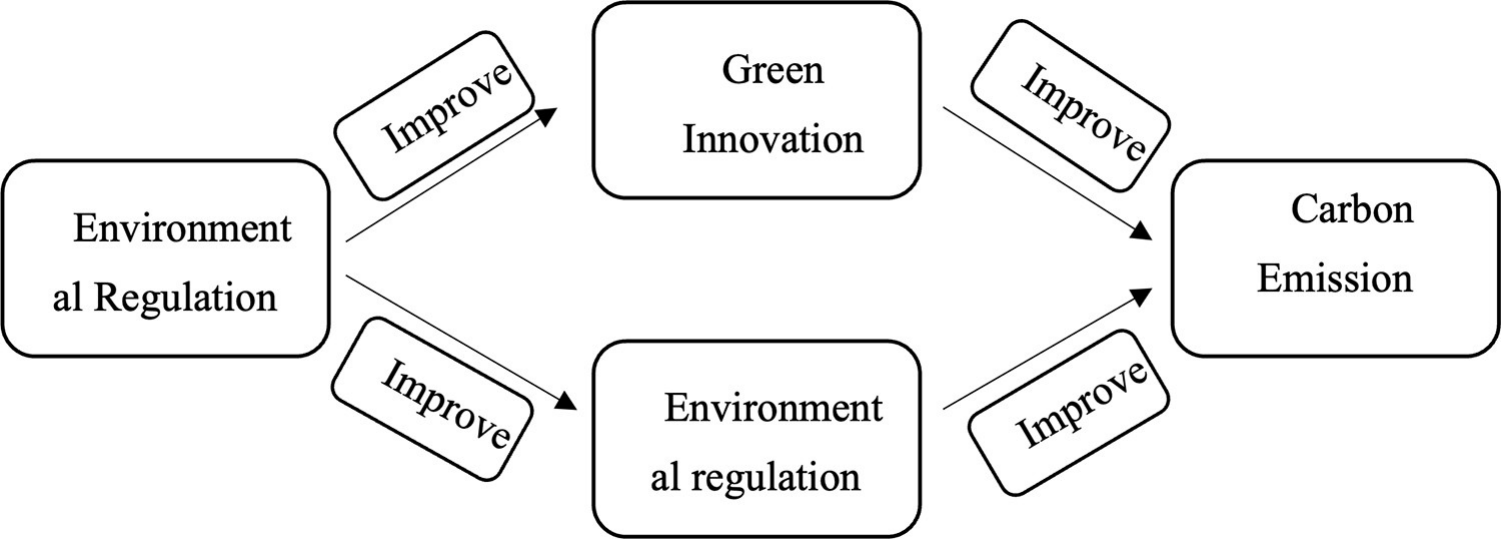
Relationship between environmental regulation and carbon emission efficiency.

To better understand the relationship between environmental regulation and carbon emission efficiency, a diagram is presented in Fig 1, which is shown as follows:

## 4. Methods and data

### 4.1. Spatial difference-in-differences model

The Pollution Levy Standards Adjustment is used as a quasi-natural experiment, and considering the spatial spillover effect [49, 50], a Spatial difference-in-differences (DID) model is employed in this paper.

To incorporate spatial factors, three methods are widely used, including Spatial Lag Model (SLM), Spatial Error Model (SEM), and spatial Durbin Model (SDM) [50], but they are slightly different, the spatial lag terms are used in SLM, the spatial lag error terms are incorporated in SEM, SDM combines both SLM and SEM. To ensure the robustness, all three models are applied.

First, SLM specification is given below:

$$Y_{it}=\alpha +\delta \sum_{j=1}^{n}W_{ij}Y_{it}+\beta {PLSA}_{it}+\varepsilon_{it},\varepsilon_{it}∼N(0,\sigma^{2}I)$$ (1)

where Y_it_ is the carbon emission efficiency, PLSA_it_ denotes implementation of PLSA, $\delta \sum_{j=1}^{n}W_{ij}Y_{it}$ denotes the spatial lag terms.

Second, the SEM specification is given below:

$$Y_{it}=\alpha +\beta {PLSA}_{it}+\varepsilon_{it}$$ (2)

$$\varepsilon_{it}=\lambda W_{it}\varepsilon +\mu ,\mu ∼N(0,\sigma^{2}I)$$ (3)

where λ denotes the coefficient of spatial auto-correlation error terms.

Finally, the SDM model is specified as:

$$Y_{it}=\alpha +\delta \sum_{j=1}^{n}W_{ij}Y_{it}+\beta {PLSA}_{it}++\xi \sum_{j=1}^{n}W_{ij}{PLSA}_{it}+\lambda {Con}_{it}$$

$$+\tau \sum_{j=1}^{n}W_{ij}{Con}_{it}+\varepsilon_{it},\varepsilon_{it}∼N(0,\sigma^{2}I)$$ (4)

where Con_it_ is the control variables; $\sum_{j=1}^{n}W_{ij}Y_{it},\;\sum_{j=1}^{n}W_{ij}{PLSA}_{it}$, and $\sum_{j=1}^{n}W_{ij}{Con}_{it}$ denote the spatial lag terms of carbon emission efficiency, PLSA, and control variables, respectively. According to current literature [51], due to spatial hysteresis, the point estimation may be biased to estimate the spatial spillover. Thus, the total effect can be divided into direct effect and indirect effect with calculus method.

The SDM model can be transformed into the following equation:

$$Y_{t}=(1-\delta W{)}^{-1}(\beta {PLSA}_{t}+\gamma W{PLSA}_{t})+(1-\delta W{)}^{-1}\varepsilon_{t}$$ (5)

The equation above can be expressed by a partial differential matrix, more specifically, taking the k-th independent variable as the example:

$${\left[\begin{array}{ccc} \frac{\partial Y}{\partial X_{1k}} & \cdots & \frac{\partial Y}{\partial X_{NK}} \end{array}\right]}_{t}=(1-\delta W{)}^{-1}\left[\begin{array}{cccc} \beta_{k} & W_{12}\lambda_{k} & \cdots & W_{1N}\lambda_{k}\\ W_{21}\lambda_{k} & \beta_{k} & \cdots & W_{2N}\lambda_{k}\\ \vdots & \vdots & \ddots & \vdots \\ W_{N1}\lambda_{k} & W_{N2}\lambda_{k} & \cdots & \beta_{k} \end{array}\right]$$ (6)

From Eq 6, the mean of diagonal elements and off-diagonal elements are shown in the partial differential matrix, respectively. The changes of the independent variables in this region denote the direct effect, and the changes in other regions present the indirect effect.

### 4.2. Measurement of variables

#### 4.2.1. Carbon emission efficiency

As we discussed above, the extended SFA model is used to measure carbon emission efficiency [15]. The model specification is given below:

$${CE}_{i,t}=\beta_{0}+f(X_{i,t};\beta )+\mu_{it}+\lambda_{i}-\tau_{it}-\gamma_{i}$$ (7)

$$RCEE_{i,t}=\exp(-{\hat{\tau }}_{it})$$ (8)

$$PCEE_{i}=\exp(-{\hat{\gamma }}_{i})$$ (9)

$$CEE_{i,t}=PCEE_{i}\times RCEE_{i,t}$$ (10)

where CE_i,t_ presents city-level carbon emission, f(X_i,t_; β) is the random frontier function with output determinants X_i,t_ [17, 52], μ_it_ denotes the random errors, λ_i_ is the city effect. The inefficiency of continuous, and residual carbon emissions are presented by τ_it_ and γ_i_, respectively. In addition, the random terms are assumed to be normally distributed as: $\mu_{it}∼N(0,\sigma_{u}^{2})$, $\lambda_{i}∼N(0,\sigma_{\lambda }^{2})$, $\tau_{it}∼N^{+}(0,\sigma_{\tau }^{2})$, $\gamma_{i}∼N^{+}(0,\sigma_{\gamma }^{2})$. The persistent carbon emission efficiency (PCEE) is determined by Eq 9, and Eq 10 presents the residual carbon emission efficiency (RCEE). The carbon emission efficiency (CEE) is obtained by the product of PCEE and RCEE.

#### 4.2.2. Environmental regulation

In this paper, the environmental regulation is measured by the implementation of Pollution Levy Standards Adjustment (PLSA), more specifically, PLSA equals 1 if the observation is after the implementation of PLSA, otherwise it is 0.

Following current literature [53–60], numerous control variables are added in our model specification, including the gross domestic product (Gdp), industrial structure (Is), government intervention (Gov), financial development (Fin), and foreign direct in-vestment (Fdi). The detailed definition of variables is shown in Table 2.

**Table 2:** Variable definition.

Classification	Symbol	Definition	Measurement
Dependent variable	CEE	Carbon emission efficiency	Defined as Eq 10
Independent variables	PSLA	Pollution Levy Standards Adjustment	PLSA equals 1 if the observation is after the implementation of PLSA, otherwise it‘s 0.
Control variables	Gdp	Gross domestic product	Natural logarithm of gross domestic product per capita
	Is	Industry structure	Output of tertiary industry divided by output of secondary industry
	Gov	Government intervention	Government expenditure scaled by gross domestic product
	Fin	Financial development	Deposit and loan balance of financial institutions scaled by gross domestic product
	Fdi	Foreign direct investment	Foreign direct investment scaled by gross domestic product
Variables in KLH-SFA	∅lngdp	Economic aggregate	Natural logarithm of gross domestic product
	∅lnpop	Total population	Natural logarithm of population
	∅lngov	Government expenditure	Natural logarithm of government expenditure
	∅lnind	Total industrial output	Natural logarithm of total industrial output

### 4.3. Data

The dataset is annual and covers 2004–2015. The carbon emission sample is extracted from the Open-source Data Inventory for Anthropogenic CO2 (ODIAC) [61], other data are obtained from two database: include the Chinese City Statistics Database (CCSD) in Chinese Research Data Services (CNRDS) Platform and the China Urban Statistical Yearbook. Missing values are excluded to form a balanced panel. Finally, our sample is reduced to 238 cities with 2856 observations. The descriptive statistics are presented in Table 3, the carbon emission efficiency has an average of 0.497 with a standard deviation of 0.169.

**Table 3:** Descriptive statistics.

Variable	N	Mean	Std	Min	Median	Max
CEE	2,856	0.497	0.169	0.071	0.504	0.818
PLSA	2,856	0.367	0.482	0.000	0.000	1.000
Gdp	2,856	10.552	0.643	4.605	10.538	13.056
Is	2,856	0.488	0.096	0.177	0.488	0.851
Gov	2,856	0.172	0.078	0.044	0.157	1.485
Fin	2,856	0.906	0.564	0.112	0.724	6.071
Fdi	2,856	0.003	0.003	0.000	0.002	0.030
∅lnco2	2,856	16.877	0.920	13.795	16.848	19.422
∅lngdp	2,856	16.513	0.912	14.067	16.414	19.605
∅lnpop	2,856	14.669	0.820	12.387	14.633	18.241
∅lngov	2,856	5.962	0.641	3.833	5.981	8.131
∅lnind	2,856	15.774	0.940	12.863	15.729	18.417

## 5. Empirical results

### 5.1. Spatial autocorrelation test

**Fig 2:**
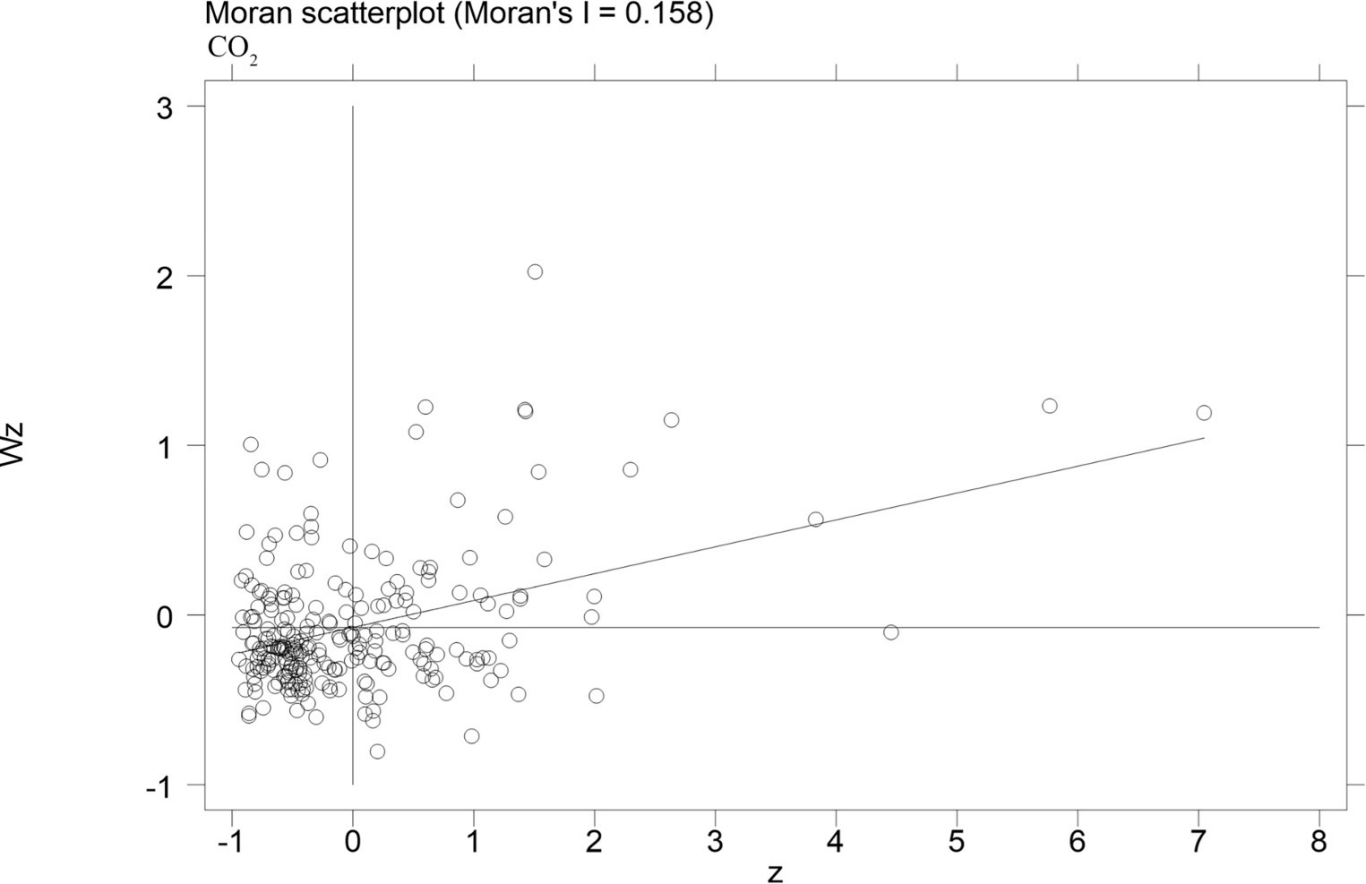
Moran scatterplots of urban carbon emission efficiency in 2004.

**Fig 3:**
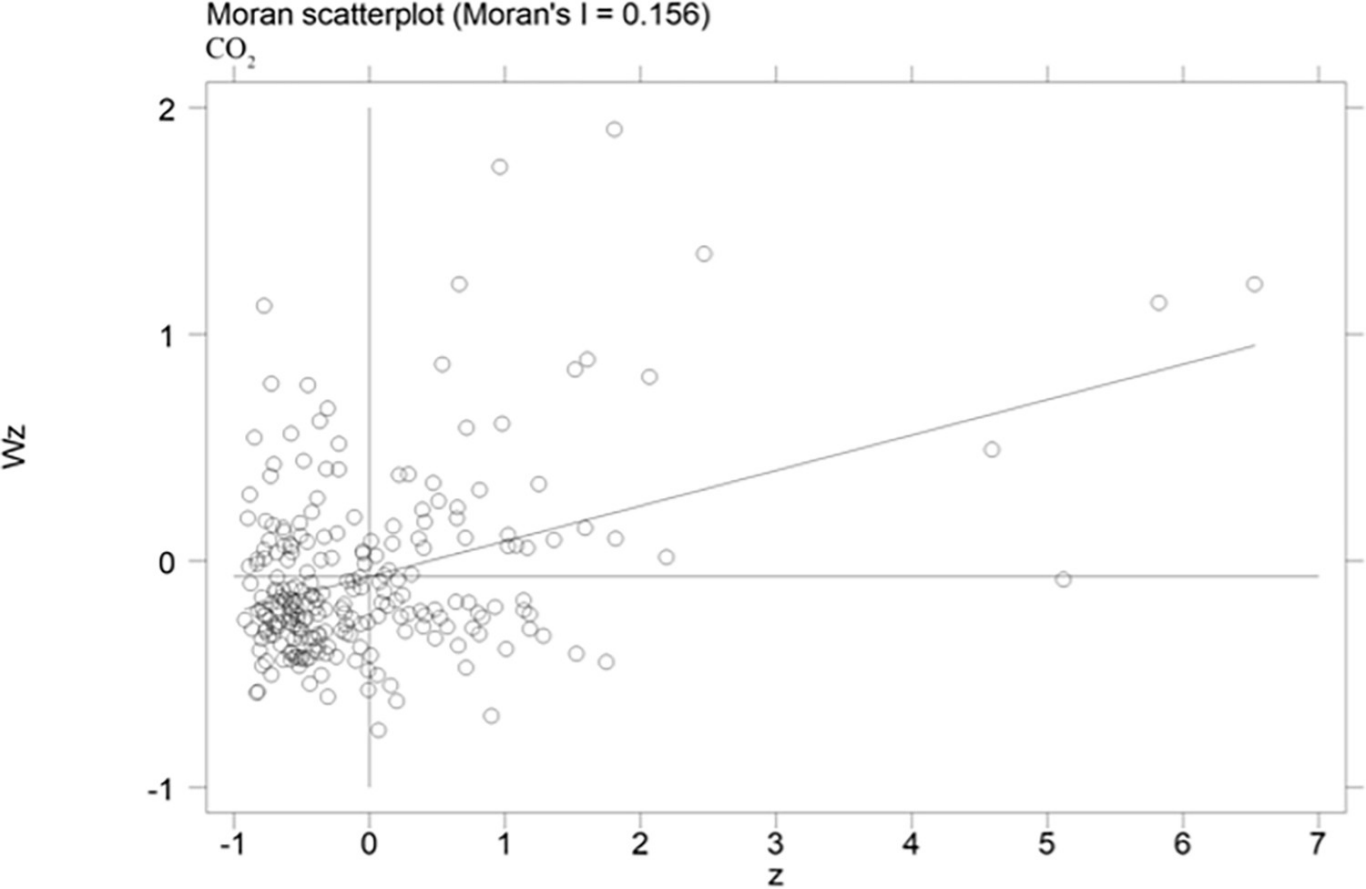
Moran scatterplots of urban carbon emission efficiency in 2009.

**Fig 4:**
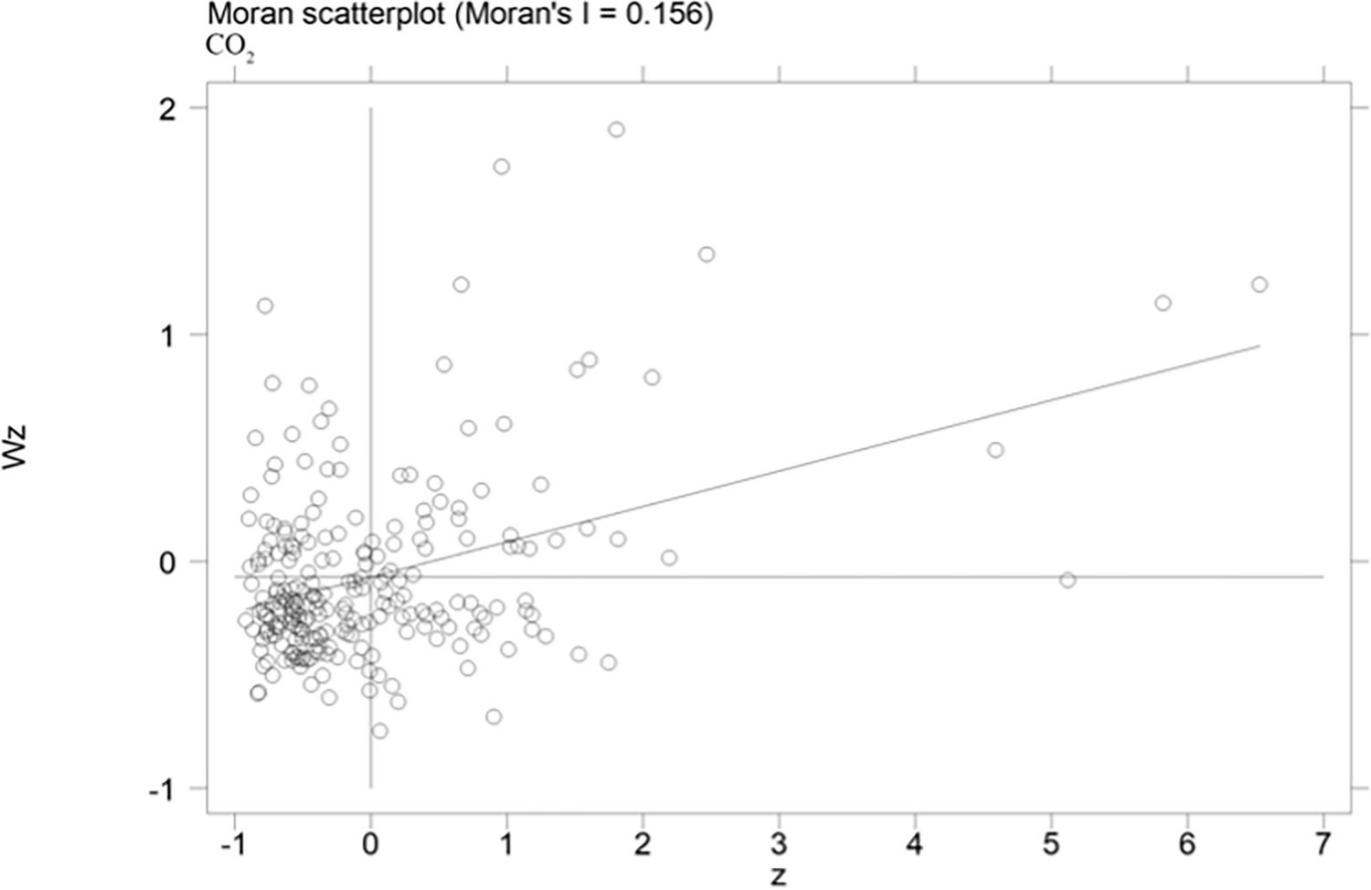
Moran scatterplots of urban carbon emission efficiency in 2015.

The spatial correlation of carbon emission efficiency of cities is computed in this paper, and scatterplots of Moran index can reflect the spatial correlation of carbon emission efficiency more visually. The scatterplots of carbon emission efficiency of cities for the years of 2004, 2009 and 2015 are presented in Figs 2–4, respectively. More specifically, the horizontal axis shows the standardized carbon emission efficiency, and the vertical axis presents the spatial lagged values. The coefficients of the primary fit line are significantly positive, it indicates that there is a spatial positive correlation between the urban carbon emission efficiency. Table 4 shows the Moran index of carbon emission efficiency, the Moran index is significantly positive at the 1% level from 2004 to 2015. The results show that the carbon emission efficiency of cities in China has a strong spatial correlation. Therefore, spatial factors should be considered in the estimation model.

**Table 4:** Moran’s I index of urban carbon emission efficiency.

	Moran’s I	Z-value
2004	0.158***	4.937
2005	0.162***	5.041
2006	0.156***	4.852
2007	0.156***	4.859
2008	0.156***	4.862
2009	0.156***	4.863
2010	0.156***	4.866
2011	0.156***	4.862
2012	0.156***	4.867
2013	0.156***	4.859
2014	0.156***	4.859
2015	0.156***	4.858

Note

***, **, and * denote significant at the 1% level, 5% level and 10% level.

### 5.2. Baseline regressions

Table 5 reports the baseline regression results. The results of the fixed effects model are presented in column 1, column 2 shows the results of the SLM model, the results of SEM model and the SDM model are presented in columns 3 and 4 respectively. City and year fixed effects are controlled for all specifications.

**Table 5:** Environmental regulation and carbon emission efficiency.

	FE	SLM	SEM	SDM
	(1)	(2)	(3)	(4)
PLSA	0.012***	0.019***	0.016***	0.016***
	(0.002)	(0.002)	(0.002)	(0.002)
Gdp	-0.015***	-0.003***	-0.009***	-0.013***
	(0.002)	(0.001)	(0.002)	(0.002)
Is	-0.081***	-0.020***	-0.061***	-0.043***
	(0.009)	(0.008)	(0.008)	(0.009)
Gov	-0.088***	-0.044***	-0.067***	-0.080***
	(0.009)	(0.010)	(0.010)	(0.010)
Fin	0.010***	0.008***	0.011***	0.009***
	(0.002)	(0.002)	(0.002)	(0.002)
Fdi	-0.026	0.049	0.084	0.155
	(0.186)	(0.201)	(0.192)	(0.192)
wSFP				-0.020***
				(0.005)
wGdp				0.026***
				(0.003)
wIs				0.133***
				(0.016)
wGov				0.098***
				(0.024)
wFin				-0.019***
				(0.004)
wFdi				-0.975**
				(0.460)
City FE	Y	Y	Y	Y
Year FE	Y	Y	Y	Y
Obs	2856	2856	2856	2856
R^2^	0.392	0.047	0.065	0.098

Note

***, **, and * denote significant at the 1% level, 5% level and 10% level. City-level cluster robust standard errors are reported in parentheses.

From Table 5, our empirical results show that the coefficients of PLSA are significantly positive for all specifications, it indicates that the Pollution Levy Standards Adjustment policy significantly improves the carbon emission efficiency. Specifically, the implementation of PLSA can induce approximately 2% increase in carbon emission efficiency. The empirical results coincide with our expectation that environmental regulation will improve carbon emission efficiency.

Moreover, the coefficients of Gdp and Is are significantly negative, indicating that carbon emission efficiency increases with economic growth. Higher GDP and Is present higher economic growth, and it will lead to higher energy consumption, which further result in higher carbon emission, thus, carbon emission efficiency will be reduced. The coefficient of Gov is negative as well, thus, the government expenditure will reduce the carbon emission efficiency. In addition, the coefficients of Fin are significantly positive, it indicates that more developed financial institutions induce more efficient carbon emission. However, the coefficient of Fdi is insignificant.

The estimation results of SDM model show that the implementation of PLSA enhances carbon emission efficiency. Since PLSA pilot cities are distributed across the country and carbon emission efficiency is also spatially correlated, it is necessary to discuss the spatial spillover effects. Table 6 further reports the spatial spillover effects of PLSA on carbon emission efficiency. Specifically, the direct, indirect, and total effects of PLSA on carbon emission efficiency improvement are all significantly positive at the 1% level, thus, PLSA in the city can significantly improve the carbon emission efficiency of this city at 1.7%, and it also significantly improves the carbon emission efficiency of other cities at 3.7%.

**Table 6:** Direct effect, indirect effect, and total effect of SDM in Table 5.

	PLSA	Gdp	Is	Gov	Fin	Fdi
Direct effect	0.017***	-0.012***	-0.036***	-0.077***	0.009***	0.119
	(0.002)	(0.002)	(0.009)	(0.009)	(0.002)	(0.198)
Indirect effect	0.037***	0.031***	0.174***	0.104***	-0.023***	-1.321*
	(0.007)	(0.003)	(0.021)	(0.034)	(0.007)	(0.688)
Total effect	0.054***	0.019***	0.138***	0.027	-0.014**	-1.202
	(0.008)	(0.003)	(0.021)	(0.037)	(0.007)	(0.767)

Note

***, **, and * denote significant at the 1% level, 5% level and 10% level. City-level cluster robust standard errors are reported in parentheses.

### 5.3. Parallel trend test

**Fig 5:**
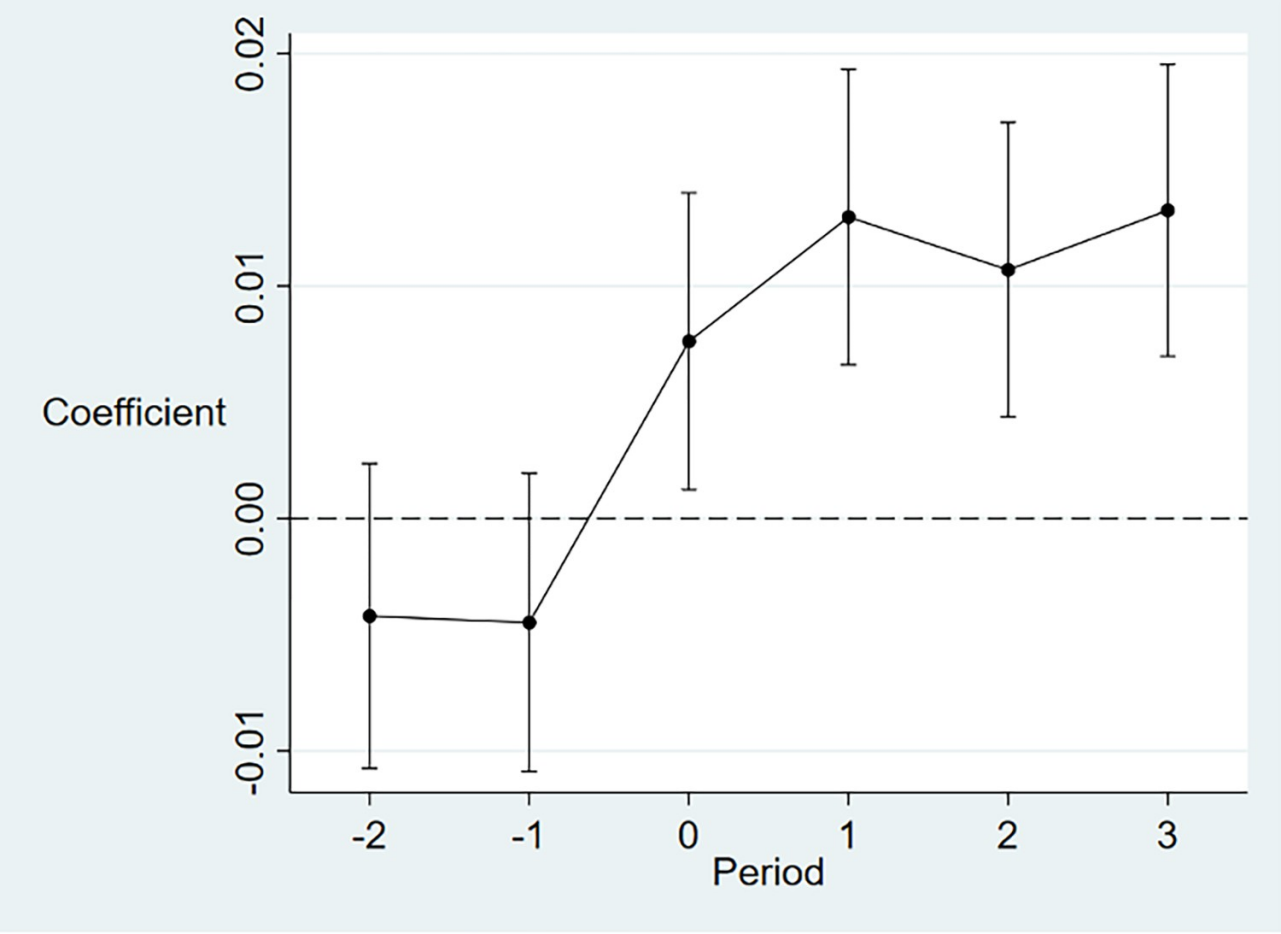
Results of parallel trend test. The X-axis denotes the window period for PLSA implementation. The Y axis represents the regression coefficient of PLSA implementation. The year before PLSA is implemented as the base period.

The results of the parallel trend test are reported Fig 5. It can be seen that both of the coefficients for two years and one year before the implementation of PLSA are statistically insignificant, this evidence indicates the satisfaction of the parallel trend. In addition, the coefficients for one year, two years, and three years after the PLSA implementation are positive and significant, it suggests that carbon emission efficiency improves after the enforcement of PLSA, and the improvement maintains thereafter.

### 5.4. Further analysis

#### 5.4.1. Impact mechanism of green innovation and industrial upgrading

To test the impact mechanism, this paper constructs the following mediating effect model to explore the mechanism of the innovative city pilots affecting urban carbon emission efficiency:

$${CEE}_{it}=\alpha_{0}+\alpha_{1}{PLSA}_{it}+Controls+\varepsilon_{it}$$ (11)

$${Mv}_{it}=\beta_{0}+\beta_{1}{PLSA}_{it}+Controls+\varepsilon_{it}$$ (12)

$${CEE}_{it}=\gamma_{0}+\gamma_{1}{PLSA}_{it}+{\gamma_{2}Mv}_{it}+Controls+\varepsilon_{it}$$ (13)

where Mv_it_ denotes the mediating variable, including green innovation (Green_inn) and industrial upgrading (Iu). Specifically, green innovation is measured by the logarithm of the total number of green invention patents and green applicable patents in city i of year t [62, 63]. Following current literature [64], industrial upgrading is measured as:

$${Iu}_{it}=\sum_{m=1}^{3}y_{imt}\times m,m=1,2,3$$ (14)

r denotes the evolution of the proportional relationship between the three major industries in China from the dominance of the primary industry to the dominance of the secondary and tertiary industries. Larger Iu indicates higher industrial upgrading. Table 7 reports the empirical results for mediation effects, columns 1 and 2 show the green innovation channel, and the industrial upgrading channel is reported in columns 3 and 4, city and year fixed effects are added for all specifications.

**Table 7:** Impact mechanism of green innovation and industrial upgrading.

	Green_inn	Cee	Iu	Cee
	(1)	(2)	(3)	(4)
PLSA	1.396***	0.011***	0.197***	0.013***
	(0.105)	(0.002)	(0.018)	(0.002)
Green_inn		0.008***		
		(0.001)		
Iu				0.032***
				(0.004)
C	6.734***	0.636***	6.870***	0.501***
	(0.069)	(0.020)	(0.012)	(0.033)
Control	Y	Y	Y	Y
City FE	Y	Y	Y	Y
Year FE	Y	Y	Y	Y
Observation	2856	2856	2856	2856
F	176.798	102.498	123.482	96.204
R^2^	0.063	0.416	0.045	0.387

Note

***, **, and * denote significant at the 1% level, 5% level and 10% level. City-level cluster robust standard errors are reported in parentheses.

For green innovation channel, the coefficient of PLSA in column 1 and the coefficient of Green_inn in column 2 are both statistically significant, it indicates the green innovation channel is significant. Moreover, both coefficients are positive, thus, the implementation of PLSA stimulates green innovation, and carbon emission efficiency increases with the green innovation, which coincides with our first hypothesis. In addition, the coefficient of PLSA is statistically significant in column 2, it indicates that the mediation effect of green innovation is partial.

For industry upgrading channel, the coefficient of PLSA in column 3 and the coefficient of Iu in column 4 are both statistically significant, it indicates the industry up-grading channel is significant. Moreover, both coefficients are positive, thus, the implementation of PLSA stimulates industry upgrading, and carbon emission efficiency increases with industry upgrading, which coincides with our second hypothesis. In addition, the coefficient of PLSA is statistically significant in column 4, it indicates that the mediation effect of industry upgrading is partial.

Overall, the green innovation increases with the PLSA implementation, and the increased green innovation improves carbon emission efficiency. moreover, the industry upgrading increases with the PLSA implementation, and the increased industry up-grading improves carbon emission efficiency. Both channels will lead to the positive correlation between carbon emission efficiency and the implementation of PLSA.

#### 5.4.2. Heterogeneity analysis

In terms of city heterogeneity, two features are considered: size (Big) and resource (Res). In China, the economy of larger city is more developed, small cities are less developed [65]. Then, larger cities are featured with more complex and well-developed industrial system, then, firms with advanced technology will prefer to be located in larger cities, qualified workers also tend to move to larger cities. Thus, the agglomeration of firms and workers will form the scale effect, as a result, the scale effect may differ across different cities [55]. Moreover, compared with resource-based cities, non-resource-based cities consume less energy and have lower upside of carbon emission efficiency [66, 67]. To measure size, Big is equal to 1 if the population of this city is larger than the median of all cities, otherwise, it is 0. Moreover, Res is equal to 1 if the city is resource-based, it’s 0 if it is non-resource-based. The empirical results are reported in Table 8, column 1 shows the results for Big, and the results for Res are presented in column 2. Both city and year fixed effects are added for all specifications.

**Table 8:** Heterogeneity analysis.

	(1)	(2)
PLSA	0.011***	-0.003
	(0.003)	(0.004)
PLSA×Big	0.007***	
	(0.003)	
PLSA×Res		0.021***
		(0.005)
Gdp	-0.013***	-0.013***
	(0.002)	(0.002)
Is	-0.042***	-0.043***
	(0.009)	(0.009)
Gov	-0.080***	-0.083***
	(0.010)	(0.010)
Fin	0.009***	0.010***
	(0.002)	(0.002)
Fdi	0.150	0.211
	(0.191)	(0.191)
wPLSA	0.037***	-0.019
	(0.008)	(0.014)
wSFP×Big	-0.023***	
	(0.007)	
wSFP×Res		0.043***
		(0.015)
wGdp	0.025***	0.027***
	(0.003)	(0.003)
wIs	0.132***	0.134***
	(0.016)	(0.016)
wGov	0.097***	0.091***
	(0.024)	(0.024)
wFin	-0.019***	-0.019***
	(0.004)	(0.004)
wFdi	-0.974**	-0.884*
	(0.459)	(0.458)
City FE	Y	Y
Year FE	Y	Y
Obs	2856	2856
R^2^	0.046	0.087

Note

***, **, and * denote significant at the 1% level, 5% level and 10% level. City-level cluster robust standard errors are reported in parentheses.

The coefficient of the interaction term between PLSA and Big is significantly positive, it indicates that the impact of PLSA is more pronounced for larger cities. Since larger cities are more developed, thus, the green innovation and industry upgrading will also be more significant. In addition, the enforcement of PLSA will also be more intense in larger cities. As a result, the impact of PLSA will be more pronounced for larger cities.

Moreover, the coefficient of the interaction term between PLSA and Res is significantly positive, indicating that the impact of PLSA is greater in resource-based cities. The energy consumption and carbon emission efficiency are much higher for resource-based cities, thus, the impact of PLSA will be larger.

## 6. Robustness test

### 6.1. Placebo test

**Fig 6:**
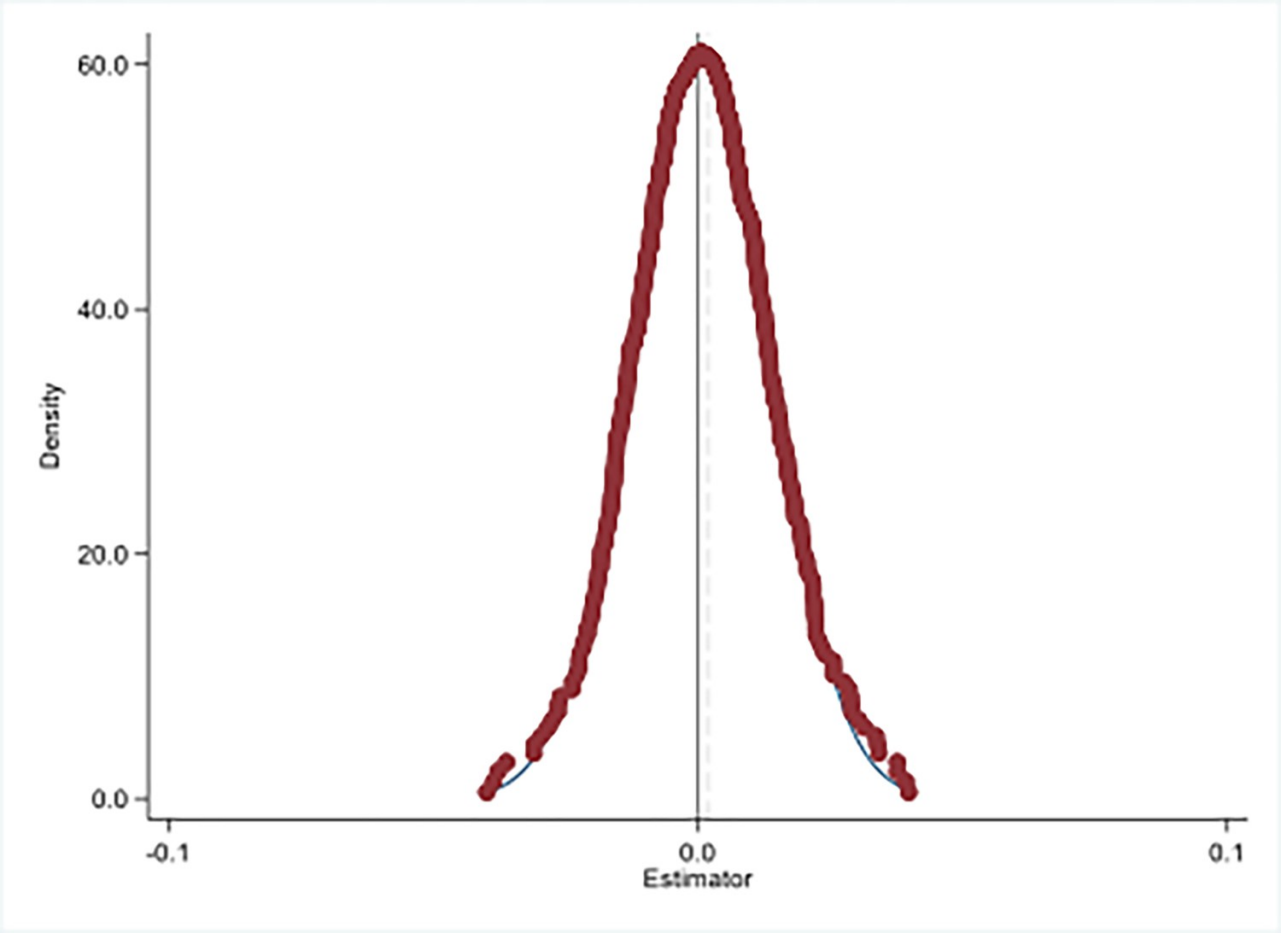
Results of placebo test. Treatment groups were randomly drawn 500 times in control group by Monte Carlo simulation and DID regression was performed. Plot the obtained regression coefficients as a distribution graph. This figure reports carbon emission efficiency of non-pilot cities as a dependent variable, presenting a normal distribution with an average value of 0.

The implementation of PLSA may also impact the carbon emission efficiency of cities without implementation of PLSA, thus, the reliability may be affected. As a result, Monte Carlo simulation is employed as our placebo test, empirical results are presented in Fig 6. We randomly draw a sample from control group to be the new treatment group, and then we re-estimate the model with DID method. If the coefficients obtained after resampling are normally distributed with a mean of 0, then the results are robust. In this paper, we randomly draw 500 times for resampling, as we expected, the re-estimated coefficients are normally distributed with zero mean. As a result, the improvement of carbon emission efficiency is originated from the PLSA.

### 6.2. Re-estimation using PSM-DID

To ensure that the sample selection bias does not affect the reliability of the conclusions in this paper, we use the difference-in-difference model after propensity score matching (PSM-DID) for re-estimation. For matching, two conventional methods are employed: namely 1:1 nearest neighbor matching and kernel density matching. The results of PSM-DID are shown in Table 8.

According to the results of Table 9, the coefficients of PLSA are 0.016 and 0.023 respectively, and both are statistically significant at 1% level. Thus, the results of PSM-DID coincide with previous findings, it indicates that the PLSA significantly improves carbon emission efficiency.

**Table 9:** Re-estimation using PSM-DID.

	Neighbor matching (n = 1)	Kernel matching
	(1)	(2)
PLSA	0.016***	0.023***
	(0.005)	(0.004)
C	0.726	-0.080
	(0.584)	(0.221)
Control	Y	Y
City FE	Y	Y
Year FE	Y	Y
Obs	469	2302
F-static	24.473	76.833
Adj-R^2^	0.383	0.321

Note

***, **, and * denote significant at the 1% level, 5% level and 10% level. City-level cluster robust standard errors are reported in parentheses.

### 6.3. Re-estimation of different dependent variable

To ensure the robustness of our model, we further use logarithm of GDP per unit of carbon emissions to measure carbon emission efficiency. Empirical results are reported in Table 10, the results of the fixed effects model are presented in column 1, column 2 shows the results of the SLM model, the results of SEM model and the SDM model are presented in columns 3 and 4 respectively. City and year fixed effects are controlled for all specifications.

**Table 10:** Re-estimation of different dependent variable.

	FE	SLM	SEM	SDM
	(1)	(2)	(3)	(4)
PLSA	0.015***	0.004***	0.023*	0.018***
	(0.003)	(0.000)	(0.002)	(0.003)
wSFP				0.070**
				(0.033)
Control	Y	Y	Y	Y
City FE	Y	Y	Y	Y
Year FE	Y	Y	Y	Y
Obs	2856	2856	2856	2856
R^2^	0.883	0.064	0.019	0.082

Note

***, **, and * denote significant at the 1% level, 5% level and 10% level. City-level cluster robust standard errors are reported in parentheses.

From Table 10, the coefficients of PLSA are significantly positive for all specifications, thus, the impact of PLSA on the alternative measure of carbon emission efficiency is also positive. The findings are consistent, the implementation of PLSA significantly improves carbon emission efficiency.

## 7. Discussions

Regarding our baseline regressions, our empirical results report that the coefficients of PLSA are significantly positive for all specifications, indicating that the implementation of Pollution Levy Standards Adjustment significantly improves the carbon emission efficiency. Specifically, the enforcement of PLSA will result in approximately 2% increase in carbon emission efficiency. Our findings coincide with current research in terms of the effect of PLSA, some scholars also prove that the imposition of pollution fees and technological innovation can help mitigate sulfur dioxide well as chemical oxygen demand emissions [68]. From the perspective of carbon emission, our results are in line with previous studies, environmental regulation can significantly reduce carbon emission [69].

Our findings prove that the implementation of PLSA stimulates green innovation, which is also in line with current studies [68]. Moreover, we also show that the increased green innovation improves carbon emission efficiency, these results coincide with prior studies in terms of the relationship between innovation and pollution, numerous scholars have analyzed the pollution abatement effect of technological innovation and found that technological innovation contributes to pollution reduction [70, 71].

Moreover, we also find that the implementation of PLSA stimulates industry upgrading, and carbon emission efficiency increases with industry upgrading, which coincides with our second hypothesis. From the perspective of environmental regulation and industry upgrading, our empirical results are in line with previous studies. Environmental regulation establishes standards to protect the environment and deal with urgent environmental issues [72]. It stimulates the development of the green industry by putting standards and restrictions on resource use, waste management, and pollution [73]. These restrictions compel industries to embrace sustainable practices, invest in cleaner technology, and create eco-friendly solutions. Implementing strict environmental regulation stimulates industry upgrading and aids in the shift to a greener economy [74].

In terms of the heterogeneity of cities, our results reveal that the impact of PLSA differs across different cities, this also has been proved in previous studies [69]. Cities with different geographical locations, economic development levels, administrative levels, levels of opening up, and environmental protection pressures have different levels of carbon emissions and carbon reduction pressures. According to the marginal effect, the carbon reduction effect in different cities is heterogeneous.

## 8. Conclusion and policy implications

### 8.1. Conclusion

For the last decades, China’s economy experienced great growth, which also induces large carbon emission. Facing the target of “Carbon peak, Carbon neutrality” in China, it is vital to improve the carbon emission efficiency.

Employing the spatial DID model, this paper investigates the impact of environ-mental regulation on carbon emission efficiency with a quasi-natural experiment of Pollution Levy Standards Adjustment in China. Our findings show that the environ-mental regulation can significantly improve the carbon emission efficiency. moreover, two impact channels are explored: green innovation and industrial upgrading. More specifically, the green innovation increases with the PLSA implementation, and the in-creased green innovation improves carbon emission efficiency. The industry upgrading increases with the PLSA implementation, and the increased industry upgrading im-proves carbon emission efficiency. Finally, in terms of city heterogeneity, we find that the impact of PLSA will be more pronounced for larger cities and resource-based cities.

The contribution of this paper consists in the following aspects. First, this paper examines the impact of environmental regulation on carbon emission efficiency, especially the environmental regulation with punishment fee, current studies generally focus on command-and-control environmental regulations. Meanwhile, we apply a quasi-natural experiment of the Pollution Levy Standards Adjustment in China for causal identification. Second, we employ spatial DID method due to spatial correlation, this method allows us to identify the spatial spillover effect. Third, we further investigate two possible impact mechanisms: green innovation channel and industry upgrading channel, and the city heterogeneity is also studied.

However, there are still some limitations in this paper. First, our data only contains city-level sample, not firm-level sample. However, firms are the entities of carbon emission, thus, the conclusion will be more precise if firm-specific data is employed. Second, for less developed cities, the enforcement of environmental regulation should be enhanced to ensure the effect of polices.

### 8.2. Policy implications

This paper highlights the important role of environmental regulation in improving carbon emission efficiency. With our findings, we propose the following policy recommendations:

First, from the results of baseline, on average, environmental regulation has significantly positive effect on improving carbon emission efficiency, thus, strengthening the implementation of environmental regulation is indispensable. Precisely, the environmental regulation studied in this paper is pollution levy, thus, environment department should strength the inspection on firm’s pollutants emission.

Second, with the implementation of pollution levy, the cost of pollutant emission will be increased. Thus, to reduce cost, firms are motivated in green innovation, they will invest more in emission reduction and other green technologies. Since innovative activities are risky and costly, governments are suggested to provides more subsidies to firms with green innovation. Government subsidies can improve the confidence of firms continuing to engage in green innovation, moreover, corporate financial constraint can be attenuated with government subsidies.

Third, due to the increased cost in pollution cost, firms in high pollution industries may leave this city and move to other cities with lower pollution levy. Thus, the government should consider more policies to attract firms from tertiary industries to invest in the cities, including tax reduction, government subsidies, etc.

Forth, from the results of heterogeneity analysis, the impact of PLSA is more pronounced for larger cities. Larger cities are featured with more complex and well-developed industrial system, then, firms with advanced technology will prefer to be located in larger cities, qualified workers also tend to move to larger cities. Thus, the agglomeration of firms and workers will form the scale effect. In this sense, to attract more qualified workers, governments should promote more policies beneficial for talents, such as housing subsidies, medical services, etc.

## Supporting information

S1 Data (XLSX)

S2 Data (DTA)
